# Surgical Volume for Intraocular Lens (IOL) Dislocation/Decentration and Associated Patient/Ocular Factors: A Retrospective Multicenter Study in Japan

**DOI:** 10.7759/cureus.98551

**Published:** 2025-12-05

**Authors:** Takahiro Usami, Hideyuki Shimizu, Tadasu Sugita, Norifumi Hirata, Maki Suzuki, Iichiro Sugita, Goichiro Miyake, Juntaro Sugita, Kensaku Miyake, Hiroki Kaneko

**Affiliations:** 1 Ophthalmology, Hamamatsu University School of Medicine, Hamamatsu, JPN; 2 Ophthalmology, Chutoen General Medical Center, Kakegawa, JPN; 3 Ophthalmology, Nagoya University Graduate School of Medicine, Nagoya, JPN; 4 Ophthalmology, Sugita Eye Hospital, Nagoya, JPN; 5 Ophthalmology, Miyake Eye Hospital, Nagoya, JPN

**Keywords:** cataract, iol decentration, iol dislocation, iol fixation, iol implantation

## Abstract

Purpose

This study aimed to analyze temporal changes in the number of surgeries performed for intraocular lens (IOL) dislocation/decentration and aphakia and to examine the associated patient and ocular background factors across three institutions.

Methods

We conducted a retrospective review of all cataract-related procedures performed between January 1, 2008, and December 31, 2014, at Nagoya University Hospital, Sugita Eye Hospital, and Miyake Eye Hospital. We quantified (1) the annual number of standard cataract surgeries (routine lens extraction with IOL implantation), (2) the annual number of IOL fixation procedures for aphakic eyes (including scleral fixation), and (3) the annual number of surgeries specifically for IOL dislocation/decentration (IOL explantation and/or IOL refixation including scleral fixation). Cases involving IOL fixation within three months after the primary cataract surgery were excluded from the IOL dislocation cohort. We also assessed the presence of diabetes, pseudoexfoliation (PEX), atopic dermatitis (AD), uveitis, and prior vitrectomy. Age and sex were also recorded. Institutional review board approvals were obtained at all three sites.

Results

The annual number of routine cataract surgeries increased significantly from 6,307 in 2008 to 8,469 in 2014 (p<0.01). Surgeries for IOL dislocation also showed a significant increase over the same period (p<0.01). In contrast, there was no significant trend in the annual number of IOL fixations for aphakia (p=0.73) or surgeries for IOL decentration (p=0.94). After Bonferroni correction, the proportions of comorbidities, including diabetes, uveitis, and prior vitrectomy, showed no significant change over time. The mean age of patients and the male-to-female ratio also remained stable throughout the study period.

Conclusions

While the volume of routine cataract surgery and procedures for IOL dislocation significantly increased, the surgical load for secondary IOL fixation in aphakic eyes remained constant. Given Japan's rapidly ageing population, there is an expected increase in the need to train surgeons who are proficient in IOL fixation, including scleral fixation.

## Introduction

Cataract surgery has advanced remarkably in recent decades, becoming a highly safe procedure due to improvements in surgical instruments and standardized techniques. In parallel, Japanese patients, even those in older age groups, exhibit high levels of activities of daily living (ADL), and indications for surgery are increasingly aligned with patient needs (e.g., driver's license renewal), thereby broadening surgical candidacy. Furthermore, the recent development of multifocal intraocular lenses (IOLs), which allow patients to choose lenses tailored to their needs, has also contributed to expanding the range of candidates for surgery [1]. Nationally, approximately 1.6 million lens reconstruction procedures are performed in Japan annually [2]. The power calculation methods for IOLs have improved year by year, and satisfaction after cataract surgery has also increased [3]. Age-related lens opacification is common, affecting around 60% of individuals in their 70s. With 25.1% of the population aged 65 years or over (according to the 2014 White Paper on the Aging Society), demand for cataract surgery is expected to increase.

Postoperative visual decline after cataract surgery is typically attributed to posterior capsule opacification and cystoid macular edema. However, more recently, there has been an increasing impression that cases of IOL decentration/dislocation are becoming more common, a view supported by previous reports [4,5]. IOL dislocation/decentration can be classified as early (≤3 months postoperatively) versus late (>3 months) [5]. Early events are mainly related to insufficient IOL stability at the time of surgery and reportedly have decreased [5]. Late events are not necessarily caused by intraoperative maneuvers; instead, progressive zonular weakness is considered a principal driver [6].

To examine whether our clinical impression was correct, we conducted a multicenter analysis across three institutions, quantifying temporal changes in surgical cases for IOL dislocation/decentration and exploring associated patient and ocular factors.

## Materials and methods

Study design

This retrospective study included cases undergoing surgery for IOL dislocation/decentration from January 1, 2008, to December 31, 2014, at three centers (Nagoya University Hospital, Sugita Eye Hospital, and Miyake Eye Hospital). We systematically reviewed the surgical records and electronic medical charts of the three participating centers. Data were extracted by trained personnel and anonymized before analysis. We first assessed annual counts of routine cataract surgery (standard lens reconstruction with IOL insertion, excluding special procedures such as IOL fixation and IOL explantation). We then tabulated annual counts of IOL fixation for aphakic eyes, including scleral fixation, and finally enumerated cases undergoing surgery specifically for IOL dislocation/decentration (IOL explantation and/or refixation including scleral fixation). The IOL fixation procedures included scleral fixation (e.g., handshake technique, Gore-Tex suture fixation), iris fixation, and anterior chamber IOL insertion. Procedures for IOL dislocation/decentration specifically included IOL explantation and/or IOL refixation (scleral or iris). In this study, IOL decentration was defined as a partial displacement of the IOL from the visual axis while the optic remained visible within the pupillary area. IOL dislocation was defined as a significant displacement of the IOL, such as luxation into the vitreous cavity or complete subluxation requiring surgical intervention. Cases receiving IOL fixation within three months after the primary cataract surgery were excluded. For comorbid conditions in eyes undergoing IOL dislocation/decentration surgeries, we recorded diabetes, glaucoma, pseudoexfoliation (PEX), atopic dermatitis (AD), uveitis, and any history of vitreous surgery. For IOL dislocation/decentration surgeries, we also noted patient age at surgery and sex. This multicenter retrospective study was approved by the Institutional Review Boards of Nagoya University Hospital, Sugita Eye Hospital, and Miyake Eye Hospital (approval numbers: 2016-0220, 401, 2016-No. 3).

Surgical techniques for IOL dislocation/decentration

The specific surgical approach for IOL dislocation/decentration (refixation or explantation) was determined by the attending surgeon based on the IOL type, the extent of dislocation, and the remaining capsular support. Scleral fixation of the IOL, which was primarily performed for eyes lacking sufficient capsular support (e.g., PEX, previous trauma), included various methods such as sutured fixation (using 9-0 or 10-0 Prolene or Gore-Tex suture) to the sclera or sutureless techniques (e.g., flanged intrascleral IOL fixation). IOL explantation was performed when the existing IOL was deemed unsuitable for refixation. Details of the surgical instruments and specific techniques followed the standard practice guidelines of each participating center.

Statistical analysis

All trends were analyzed using simple linear regression. The dependent variables for trend analysis were the annual counts of aphakic eyes and IOL fixation, IOL dislocation/decentration surgery, and routine cataract surgery. The independent variable was the year (2008-2014). A p-value of less than 0.05 was considered to be statistically significant. To evaluate the trend in the proportion of comorbidities, a Bonferroni correction was applied to account for multiple comparisons of the five defined comorbidities (diabetes, glaucoma, PEX, AD, and uveitis) against the annual surgical volume. A p-value of less than 0.01 (i.e., 0.05 divided by 5) was considered statistically significant for these specific comparisons. All statistical analyses were performed using Python Version 3.11 (Python Software Foundation, Fredericksburg, VA, USA) and R Version 4.5.1 (R Foundation for Statistical Computing, Vienna, Austria).

## Results

Annual volume of routine cataract surgery

**Figure 1:**
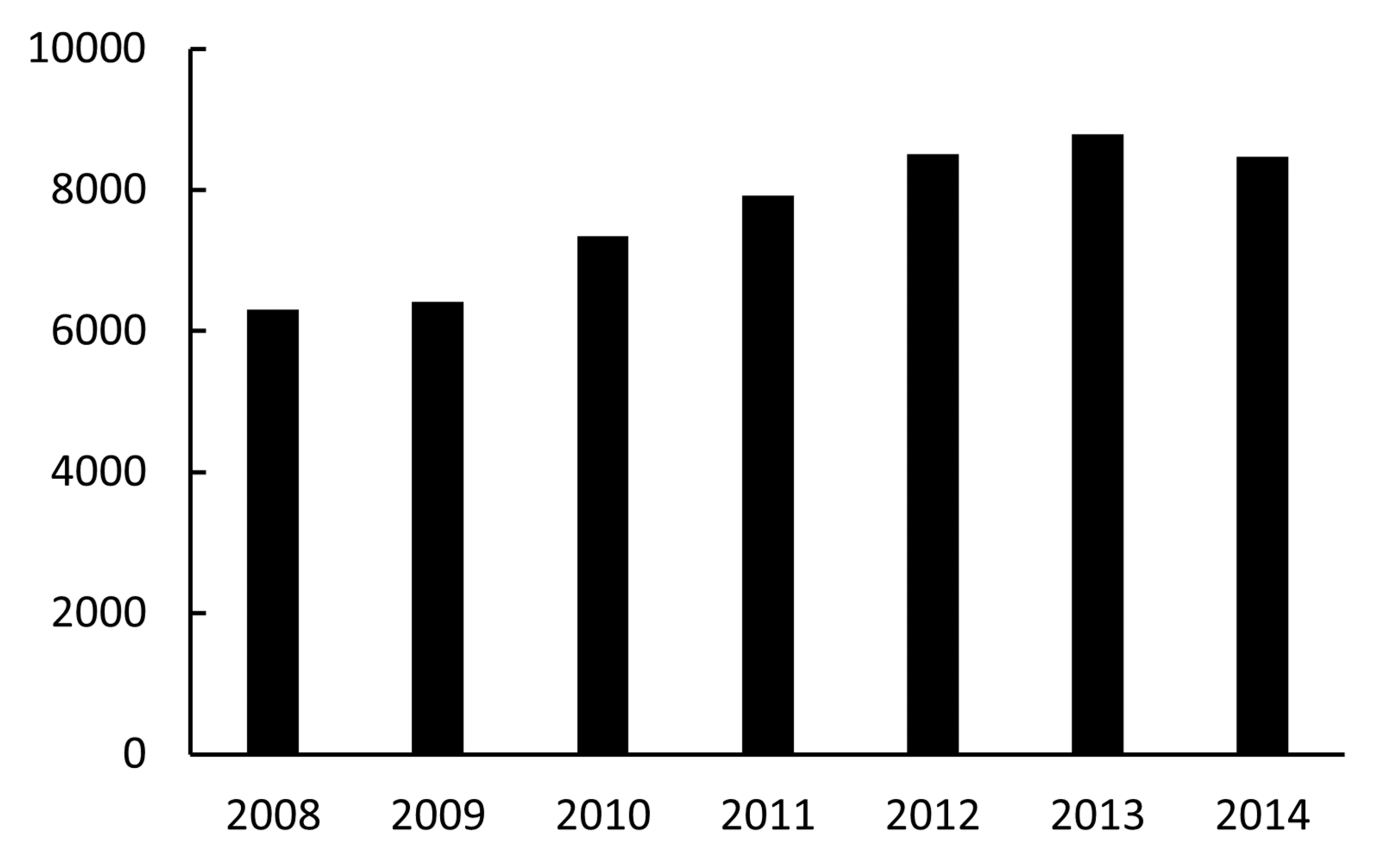
Annual volume of routine cataract surgery The X-axis and Y-axis indicate the year and the total number of routine cataract surgeries (IOL implantation), respectively. IOL: intraocular lens

The combined annual numbers of routine cataract surgeries (IOL implantation) were 6,307 in 2008, 6,416 in 2009, 7,345 in 2010, 7,924 in 2011, 8,508 in 2012, 8,792 in 2013, and 8,469 in 2014. From 2008 to 2014, the annual number of surgical procedures demonstrated a statistically significant increase. A strong, positive correlation was observed between the year and the number of surgeries performed (R^2^=0.89; p<0.01). On average, the number of procedures increased by approximately 551 cases per year during this period (Figure 1).

Aphakic eyes and IOL fixation

**Figure 2:**
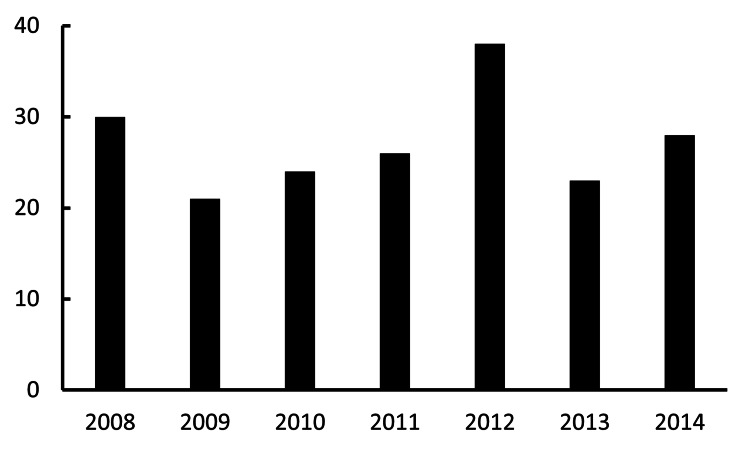
Annual volume of aphakic eyes and IOL fixation The X-axis and Y-axis indicate the year and the total number of aphakic eyes and IOL fixation surgeries, respectively. IOL: intraocular lens

Historically, aphakia arose from trauma, remote cataract extraction, or pediatric cataract. The annual numbers of aphakic eyes and IOL fixation were 30 in 2008, 21 in 2009, 24 in 2010, 26 in 2011, 38 in 2012, 23 in 2013, and 28 in 2014. There was no statistically significant increasing or decreasing trend in the annual values. The year was not a significant predictor of the outcome, as indicated by the low coefficient of determination and non-significant p-value (R^2^=0.027; p=0.73). With contemporary techniques (e.g., capsular tension ring (CTR) use, advances in phaco platforms such as the Centurion), the frequency of leaving eyes aphakic appears to have decreased or at least not increased, as more eyes receive primary IOL implantation even in settings that previously favored aphakia (e.g., shallow anterior chamber, zonular instability) (Figure 2).

IOL dislocation surgeries

**Figure 3:**
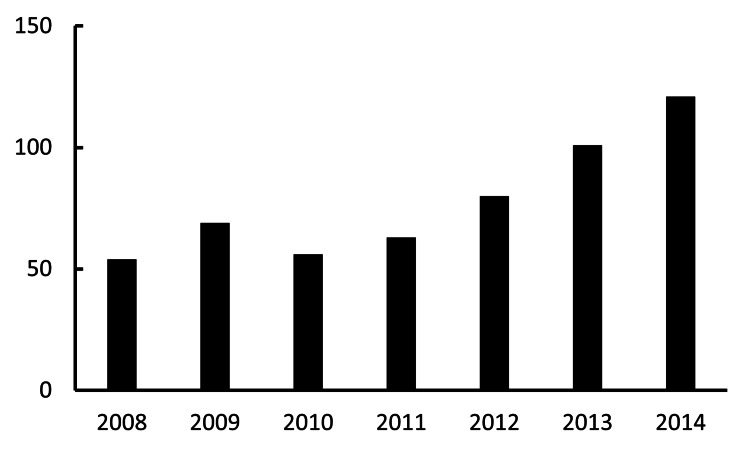
Annual volume of surgeries addressing IOL dislocation The X-axis and Y-axis indicate the year and the total number of IOL dislocation surgeries, respectively. IOL: intraocular lens

The annual numbers of IOL dislocation surgeries were 54 in 2008, 69 in 2009, 56 in 2010, 63 in 2011, 80 in 2012, 101 in 2013, and 121 in 2014. The annual number of surgical procedures demonstrated a statistically significant increase. A strong, positive correlation was observed between the year and the number of surgeries performed (R^2^=0.77; p<0.01). On average, the number of procedures increased by approximately 10.5 cases per year during this period (Figure 3).

IOL dislocation/decentration surgeries

**Figure 4:**
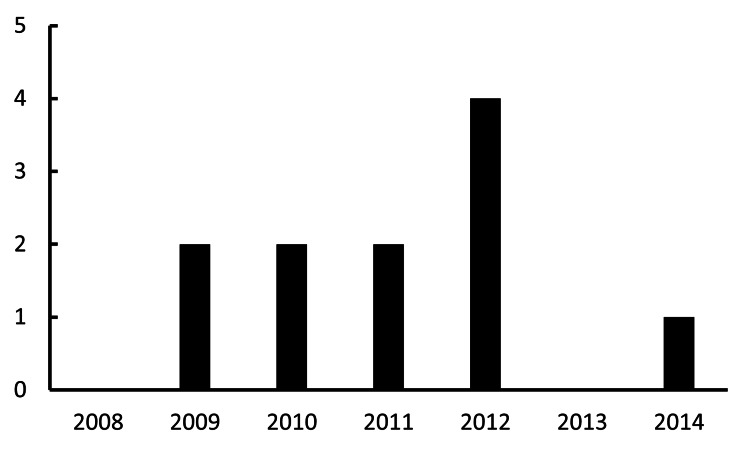
Annual volume of surgeries addressing IOL decentration The X-axis and Y-axis indicate the year and the total number of surgeries for IOL decentration, respectively. IOL: intraocular lens

The annual numbers of IOL dislocation surgeries were zero in 2008, two in 2009, two in 2010, two in 2011, four in 2012, zero in 2013, and one in 2014. There was no statistically significant trend in the annual number of deviations. No correlation was found between the year and the number of cases (R^2^=0.001; p=0.94) (Figure 4).

**Figure 5:**
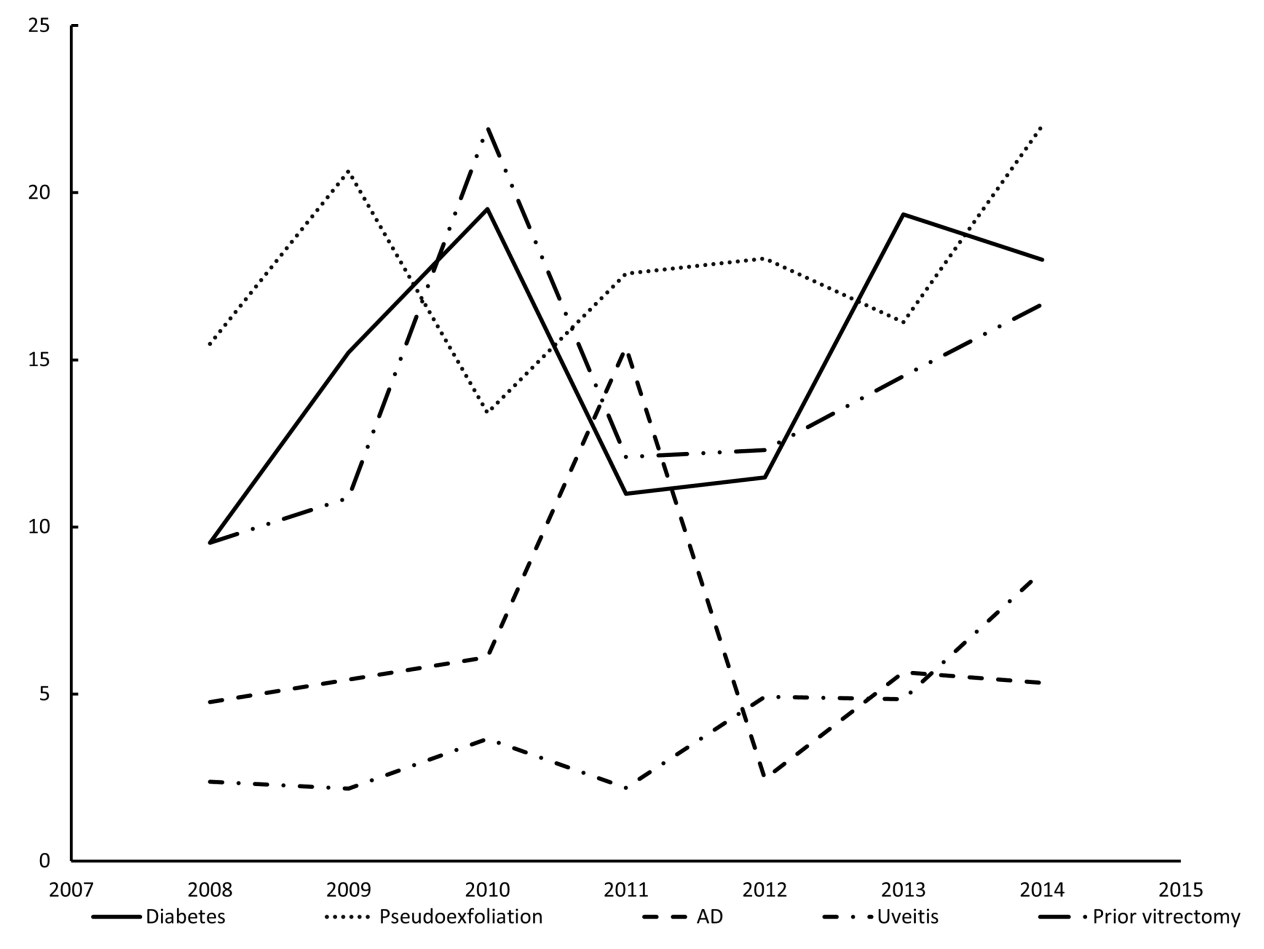
Annual comorbid conditions in eyes undergoing surgery for IOL dislocation/decentration The X-axis and Y-axis indicate the year and the proportion of each comorbidity relative to the total number of cases, respectively, analyzed separately based on their causes, i.e., diabetes, pseudoexfoliation, AD, uveitis, and previous history of vitrectomy surgeries. AD: atopic dermatitis; IOL: intraocular lens

Associated conditions among IOL dislocation/decentration cases included diabetes, PEX, AD, uveitis, and prior vitrectomy (Figure 5). After applying the Bonferroni correction for multiple comparisons, no statistically significant trend was found in the annual proportion of any of the five comorbidities (all p>0.01) from 2008 to 2014. For uveitis, which had shown a positive trend before correction (p=0.017), the result was no longer statistically significant after applying the stricter p-value threshold.

**Figure 6:**
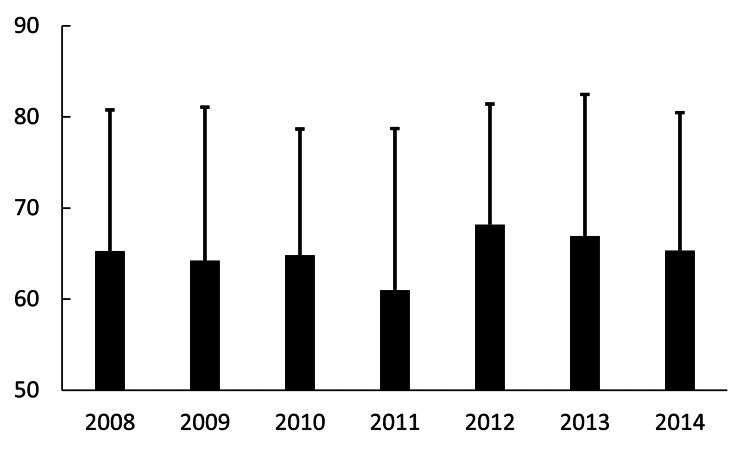
Average age at annual surgery for IOL dislocation/decentration The X-axis and Y-axis indicate the year and the patients' age (the mean age±standard deviation), respectively. IOL: intraocular lens

The mean age (±SD) among IOL dislocation/decentration surgeries was 65.2±15.4 years in 2008, 64.2±16.8 years in 2009, 64.8±13.9 years in 2010, 61.0±17.8 years in 2011, 68.1±13.2 years in 2012, 66.9±15.5 years in 2013, and 65.4±15.1 years in 2014 (Figure 6). There was no statistically significant trend in the mean age of patients undergoing surgery from 2008 to 2014. The year was not a significant predictor of the mean age, as indicated by a non-significant p-value and a low coefficient of determination (R^2^=0.006; p=0.86).

**Figure 7:**
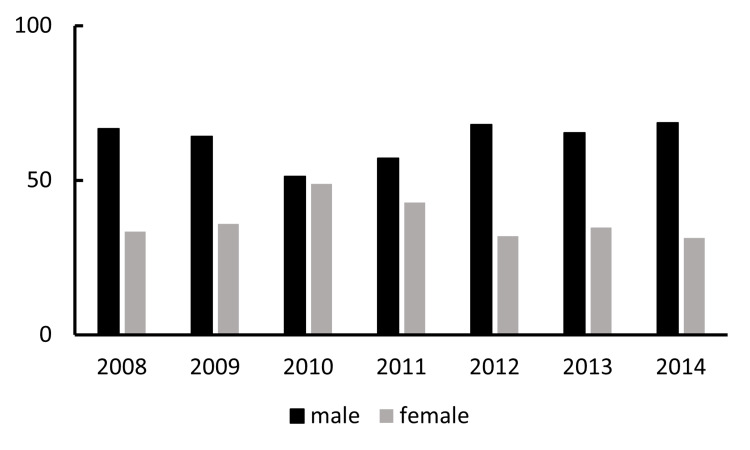
Annual percentage of patients undergoing surgery for IOL dislocation/decentration by gender The X-axis and Y-axis indicate the year and the percentage of IOL dislocation/decentration surgeries, respectively, analyzed separately based on the patients' gender. IOL: intraocular lens

The proportion of male patients was 66.7% in 2008, 64.1% in 2009, 51.2% in 2010, 57.1% in 2011, 68% in 2012, 65.3% in 2013, and 68.7% in 2014 (Figure 7). The proportion of male patients undergoing surgery for IOL dislocation/decentration exceeded that of females. There was no statistically significant trend in the proportion of male-to-female patients from 2008 to 2014 (R^2^=0.041; p=0.655). The gender ratio showed annual fluctuations without a clear upward or downward trend over the period.

## Discussion

Iwamoto et al. evaluated eyes that later developed IOL decentration/dislocation after cataract surgery, reporting a frequency of 0.098% and a mean interval of ~6 years to event; PEX was present in 38.6%, with other associations including prior laser iridotomy (LI) (11.4%), ocular contusion (11.4%), high myopia (9.1%), and AD (9.1%) [7]. In Japan, the prevalence of PEX varies by population; the Tajimi Study (≥40 years) reported 0.8% [8]. In our cohort, many primary surgeries were performed outside our institutions, limiting accurate ascertainment of PEX at the time of initial cataract surgery. Nonetheless, in some cases of IOL fixation, poor pupil dilation or pseudo-detachment material was observed at the pupillary margin even after IOL insertion, suggesting the presence of PEX. Assuming a long-term IOL dislocation/decentration frequency of ~0.1% and ~1.3 million cataract surgeries annually in Japan, roughly 1,300 IOL dislocation/decentration cases might occur per year nationwide. Earlier reports cited IOL dislocation/decentration frequencies of 0.2-3%, with a smaller share being late events [9-11]; however, those studies largely represent the early 1980s, when surgical difficulty, device precision, and perioperative care differed markedly, likely inflating early postoperative events relative to late ones [3]. More contemporary data indicate cumulative risks of IOL dislocation at 0.1% by 10 years, 0.2% by 15 years, 0.7% by 20 years, and 1.7% by 25 years [12], and surgery rates for late IOL dislocation range from 0.032% to 0.28% [13,14]. With the rising base of cataract surgeries, plus maturation of IOL refixation/scleral fixation techniques and devices, both the absolute number of IOL dislocation events and surgeries to manage them are expected to increase [15].

Notably, prior studies reported a mean age of ~71.2 years for patients undergoing surgery for IOL dislocation [15]. In this cohort, the average age for IOL fixation cases was in the 60s in every year, suggesting a possible trend toward younger patients undergoing the procedure. In our cohort, male predominance was observed, which was counterintuitive given that females undergo cataract surgery more often [16], PEX burden can be higher in females [17], and high myopia (another putative risk) is not less common in females. Considering longevity and the role of prolonged IOL in situ duration, one might expect more late dislocation in females; however, both our results and prior reports show the opposite [6,18,19]. This finding warrants further investigation to determine if there are sex-related factors influencing late-life zonular fragility that are not explained by lifespan, PEX prevalence, or myopia alone. A condition frequently highlighted in Japanese literature is AD as a comorbidity; few non-Japanese reports underscore AD similarly, and the detailed mechanistic emphasis largely comes from Japan [20].

This study has several limitations. First, many cases of IOL fixation for dislocated lenses had their primary cataract surgery performed at outside institutions, precluding the accurate estimation of the incidence of IOL dislocation/decentration in our source population. Second, the exact interval from primary lens surgery to IOL dislocation could not be reliably determined for many cases. Third, while we attempted to determine PEX in IOL fixation eyes, PEX at the time of the initial cataract surgery may have been under-recognized in the present review: mild cases without classic pupillary or anterior capsule deposits are difficult to diagnose retrospectively, and pseudophakia prevents the assessment of anterior capsular deposits. As a result, PEX frequency in our dataset is likely underestimated. Therefore, the lack of significant trends in comorbidities should be interpreted with caution. Fourth, axial length was not captured; therefore, we could not evaluate the relationship between high myopia and IOL dislocation. Future studies with complete primary surgery data, standardized PEX ascertainment, and biometric parameters are warranted.

## Conclusions

Our multicenter analysis reconfirmed that surgeries for IOL dislocation/decentration (including scleral fixation) have increased year by year, while routine cataract surgery volume rose modestly. This suggests that the complexity of cataract surgery management is growing significantly in clinical practice, particularly regarding late-onset complications. Consequently, in a super-aged society, training ophthalmic surgeons to competently perform IOL fixation will become increasingly important.
